# Characterization of halotolerant *Kushneria* isolates that stimulate growth of alfalfa in saline conditions

**DOI:** 10.1371/journal.pone.0322979

**Published:** 2025-05-07

**Authors:** Iqra Farooq, Niaz Ahmad, Cardon Porter, Rachel Smith, Thomas Scharf, Aden Cowley, Andrew Jenkins, Joshua D. Yates, Jonathon T. Hill, Brent L. Nielsen

**Affiliations:** 1 Department of Microbiology & Molecular Biology, Brigham Young University, Provo, Utah, United States of America; 2 National Institute for Biotechnology and Genetic Engineering College, Pakistan Institute of Engineering and Applied Sciences (NIBGE-C, PIEAS), Faisalabad, Pakistan; 3 Department of Cell Biology & Physiology, Brigham Young University, Provo, Utah, United States of America; Mahatma Jyotiba Phule Rohilkhand University, INDIA

## Abstract

A key barrier to crop production is soil salinity, which is a serious and growing problem world-wide due to inadequate water drainage, saline ground water, or inadequate rainfall to wash away soil salts. There is substantial promise for plant-associated microbes isolated from halophytes (salt-tolerant plants) to enhance growth of salt-sensitive crop plants in salty soils. The objective of this study was to identify salt-tolerant bacteria from native halophytes and characterize their ability to stimulate the growth of alfalfa in salty soil conditions. Several halotolerant bacteria, including *Kushneria, Halomonas,* and *Bacillus*, were identified from the rhizosphere or roots of three halophyte species (*Salicornia rubra, Sarcocornia utahensis,* and *Allenrolfea occidentalis*) in a saline area south of Utah Lake, Utah, USA. Biochemical properties, including indole acetic acid production, biofilm formation, phosphate solubilization and siderophore production activities, which have been associated with plant growth promoting (PGP) activity, were characterized for several isolates. Selected strains were screened for the ability to stimulate growth of alfalfa in controlled laboratory experiments. Among these strains, two independent isolates of the genus *Kushneria* were found to have significant growth-promoting activity for inoculated alfalfa plants grown under saline conditions (0.205 M or 1.2% NaCl) that mimic common salinity levels of affected soils. Plants inoculated with a combination of two *Kushneria* strains that have salt-tolerant PGP (ST-PGP) properties exhibited a statistically significant increase in plant growth over uninoculated plants. A GFP marker confirmed presence of *Kushneria* in the roots of inoculated plants. Bacteria with ST-PGP activity will be a key resource to facilitate increased crop yield from land affected by salinity, and the data presented here for two *Kushneria* isolates are promising.

## Introduction

Soil quality in many parts of the U.S. and worldwide is susceptible to a variety of stresses including drought, erosion, and increasing salinity due to evaporation and/or poor irrigation practices leading to accumulating presence of salts. At the same time, the human population is growing, and in many regions, high-quality agricultural land is decreasing due to the expansion of urban areas [1]. Salinity builds up in irrigated land due to soluble salts that are transported through irrigation water and remain in the soil post-evaporation, imposing ionic stress. In the absence of adequate leaching mechanisms, these salts amass to levels inhibitory to plant growth and may lead to the transformation of soils into sodic conditions. Sodicity is caused by a high buildup of sodium ions in the soil that negatively affects water permeability, leading to the degradation of soil structure, impinging upon both water retention and root penetration [2].

The growing challenge of salinization presents a serious menace to global food security, leading to the abandonment of an estimated 700,000 hectares of arable land annually [3–5]. As of 2022, salinity issues affect approximately 1 billion hectares of arable land globally, with the annual economic toll surpassing $27 billion [6]. In the United States, soil salinity translates into an annual crop production loss of $3.1 billion [7]. The reduction in crop yield induced by salinity is a universal phenomenon, with nations such as Australia experiencing losses of up to 50% in wheat yields due to the impact of salinity [8]. These factors will require new approaches to maintain adequate food supplies globally.

Salinity stress adversely affects the physiological and metabolic processes of plants through inhibiting seedling growth, reduced photosynthesis, ion toxicity, water stress, and decreased rates of lipid metabolism and protein synthesis [9]. Soil salinity, usually NaCl, may also hinder plant growth by water deficiencies and ion toxicity [10]. However, the effect on plant growth depends on the plant species and its salinity sensitivity or tolerance [11].

Alfalfa (*Medicago sativa L*.) is a perennial legume that belongs to the family *Fabaceae*, subfamily *Faboideae*. Alfalfa is a moderately salt tolerant plant among the legumes; however, its growth and yield is reduced at salinity above 2 dS m^−1^ [12]. Due to the high water demands for alfalfa production compared to other plants and widespread water shortages, the need to use wastewater or saline water for alfalfa irrigation is growing [13]. It has been suggested that alfalfa is more sensitive to salt stress during the seedling stage than at later growth stages [14]. The yield of alfalfa is frequently reduced when the salinity level approached 20 mM NaCl [15].

Salt-affected soils have detrimental effects on plant health due to high electrical conductivity (EC) caused by excess salts of various ions. Soil salinity can be caused by improper irrigation practices with water that contains relatively high concentrations of salts [16]. It was estimated that 12 million hectares of irrigated lands cannot be used due to salt stress [17]. On a global scale, it is expected that the salt stress of soil might result in the loss of more than 50% of agricultural land by the 21^st^ century [18,19]. Such soils are low in nutrient content, biomass, microbial community, and organic matter and are thus depicted as poor quality soils [20]. Each plant species exhibits its own level of tolerance to salt stress. Most agricultural plants, such as maize, soybean, and alfalfa are quite sensitive to salt. We refer to these as “glycophytes.” Some specialized plants, called “halophytes,” have evolved significant tolerance for salt.

Halophytes are plants that have adapted to grow in the presence of salinity and are found throughout the world in areas affected by high salt levels in the soil. There are three main mechanisms for halophyte tolerance to salt, including salt exclusion, salt secretion, and osmotic adjustment [21]. In some halophytes the roots create a barrier to uptake and transport of sodium and other ions (salt exclusion) by expression of specific ion transport genes and proteins within the plant [21,22]. Other halophytes form salt glands on leaf surfaces and salt ions are secreted from plant tissue into the glands [21,23]. The third mechanism is the production of compatible solutes such as proline, glycine betaine, polyphenols, and soluble sugars, in the cytosol to reduce and balance the osmotic pressure [21]. As with other plants, halophytes have an associated microbiome composed of a wide array of bacteria, fungi and archaebacteria, many of which may contribute to salinity tolerance of the plant [24].

There have been several studies on the use of salt-tolerant plant growth promoting bacteria (PGPB) to enhance plant growth in the presence of salt stress. One study showed that strains of *Bacillus amyloliquefaciens* and *Halomonas sp.* isolated from saline soil were able to promote the growth of tomato plants under salt stress conditions [25]. A strain of *Azospirillum brasilense* isolated from a salt-affected soil in Bangladesh was able to enhance the growth and salt tolerance of rice plants [26]. Another study investigated the potential of *Kushneria* as a biofertilizer for maize plants in saline soils [27]. One study evaluated the effect of inoculating alfalfa with different PGPR strains (*Pseudomonas putida, Bacillus cereus, and Azospirillum brasilense*), and grown with varying levels of salt stress ranging from 50 to 200 mM [28]. Another study investigated the effect of two PGPR strains, *Azospirillum lipoferum* and *Pseudomonas fluorescens* on the growth of alfalfa under salt stress conditions with 150 mM NaCl [29]. In similar studies, PGPR strains were found to significantly enhance the growth of alfalfa plants under salt stress compared to uninoculated controls [30,31].

The mechanisms by which ST-PGPRs enhance plant growth in the presence of salt stress are not fully understood, but several potential mechanisms have been proposed. One such mechanism is the production of compatible solutes, or osmoprotectants, which help maintain cellular water balance and prevent protein denaturation. Examples of osmoprotectants, as mentioned above, include proline, glycine betaine, and trehalose [32]. Some halophilic bacteria have been found to produce phytohormones, such as gibberellins, cytokinins, and abscisic acid, that help them grow and develop under salt stress conditions [1,33]. ACC deaminase activity is another mechanism by which some halophiles help plants cope with salt stress. ACC is a precursor of the growth-inhibiting plant hormone ethylene, and bacterial ACC deaminase inhibits ethylene formation [34]. Another mechanism is exopolysaccharides (EPS), these are complex sugars produced by some halophiles that can help protect the plant cells from salt stress [35,36].

Phosphorus is a crucial nutrient for plants, but its availability is limited as plants can only absorb mono- and dibasic phosphate, the soluble forms of phosphate [37]. Several soil microbial species, including *Bacillus megaterium, B. subtilis,* and *B. sircalmous* have been identified as effective phosphate solubilizers [38,39], whereas *P. fluorescens*, *P. cepacia, Burkholderia cepacia* and *Erwinia herbicola*, are reported as efficient producers of gluconic acid, which is the most frequent agent in mineral phosphate solubilization [40–42]. Iron is an essential nutrient for plants, but its abundance in soils is often unavailable for plant uptake. Many rhizosphere bacteria produce siderophores such as hydroximates and catechols (functional groups), which are optimal for binding iron [43]. Rhizosphere bacteria possess the potential to produce various phytohormones, including auxins, cytokinins, ethylene, gibberellins, and abcisic acid, which can mediate important plant growth and developmental processes such as cell division, enlargement, extension, and root development [44]. For instance, indole-3-acetic acid (IAA) or auxin-producing rhizobacteria can increase plant growth and root development, while cytokinin influences physiological and developmental processes such as cell division, root hair formation, and shoot initiation [45–47].

Several species of plant growth-promoting rhizobacteria (PGPR) have been found to stimulate plant growth under a variety of conditions [48–50]. One well-studied example is *Bacillus subtilis* (GB03), a rhizosphere bacterium that stimulates the growth of white clover (a legume) under saline conditions [51,52]. This bacterial strain produces volatile compounds that enhance plant photosynthetic capacity and chlorophyll content, induces increased endogenous sugar content, and suppresses abscisic acid (ABA)-induced RNA transcripts. Other effects include alterations in plant gene expression such as upregulating expression of the HKT1 sodium transporter gene in shoots and downregulating its expression in roots, resulting in lower sodium accumulation throughout the plant [53,54].

Bacteria from the gram-negative *Kushneria* and *Halomonas* genera have been found in association with halophytic plants and shown to enhance plant growth in saline soil [55]. *Kushneria*, a genus within the *Halomonadaceae* family, is a halotolerant bacterial group with orange pigmentation that is adapted to high salt concentrations [56]. *Kushneria* exhibits a remarkable ability to thrive in environments with up to 25% NaCl concentration, surpassing seawater salinity [56]. Its resilience in hypersaline conditions has led to its application in bioremediation for saline soils and the cultivation of salt-tolerant crops [56]. *Kushneria* strains have been isolated from diverse environments, including solar salterns, black mangrove leaves, seawater, salt mines, cured meats, and salt-fermented foods [57–63]. *Kushneria* strains have been found in both the rhizosphere and endosphere of halophytes and exhibit the capacity to produce osmolytes, bioactive compounds (such as betaine and ectoine), and plant growth hormones [59–64]. Studies have highlighted the potential of *Kushneria* in promoting plant growth and other functions as biofertilizers and phytoremediation, particularly in saline agricultural soils [65]. *Kushneria* sp. NRCC 31399 has been identified to enhance rice growth under saline conditions [66]. As a genus, *Kushneria* is recognized for its ability to accumulate compatible solutes, and some strains demonstrate antimicrobial activity against pathogenic bacteria and fungi, suggesting potential applications in antibiotic and antifungal agent development [67]. The promising applications of *Kushneria* underscore the need for continued research and exploration of this salt-tolerant bacterial genus for field application.

While we previously reported the identification of some halotolerant bacterial strains from the rhizosphere of halophyte species in a saline area south of Utah Lake [68], we have continued to screen more isolates to identify additional strains with greater salt-tolerant plant growth- promoting (ST-PGP) function, and to more fully characterize promising strains for properties that may contribute to PGP activity. In the earlier study, two strains (*Halomonas* sp. and *Bacillus* sp.) were identified that when introduced to young seedlings significantly stimulated the growth of alfalfa in the presence or absence of 0.17M-0.2M NaCl [68], conditions that reflect salt levels that are found in many salinity-affected areas. Alfalfa was chosen as the host plant as it is the fourth largest value crop in the USA and is a major crop in the Intermountain West. We report here the identification of additional bacterial strains, some of which have ST-PGP activity when used to inoculate alfalfa plants grown under saline conditions. We have focused on two *Kushneria* strains that show promise in alfalfa growth stimulation studies in greenhouse and growth chamber trials in saline soil conditions, and their potential PGP properties have been characterized.

## Materials and methods

### Bacterial strains used in this study

**Fig 1:**
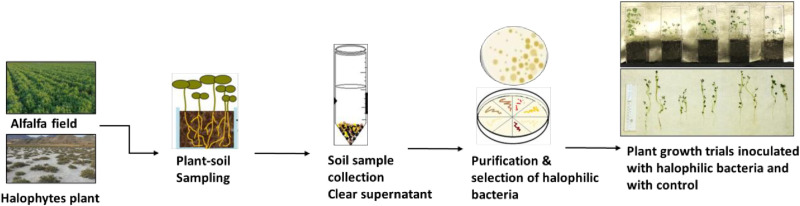
A graphical representation of isolation, screening of halotolerant bacteria and their effect on alfalfa plant growth under salt stress.

One hundred bacterial isolates were collected from halophyte plants (root and soil rhizosphere samples of *Salicornia rubra, Sarcocornia utahensis,* and *Allenrolfea occidentalis*) growing in a highly saline area near Goshen, Utah as previously described [68]. Initial isolation was performed on Luria Bertani (LB) agar plates containing 1M NaCl using serial dilution. Pure colonies were isolated and stock cultures were stored at -80°C. DNA was recovered from each isolate as described [68] and used as a template for PCR amplification of a portion of the 16S rRNA gene. PCR products were subjected to Sanger sequencing, and the results were used in NCBI BLAST searches to determine genus/species of the isolates. The orange pigmentation of colonies was also used to help confirm the identity of *Kushneria* isolates, while other strains had creamy or white colonies. The flowchart in Fig 1 illustrates the steps involved in this study.

### *In-vitro* screening of halotolerant bacteria for the detection of plant growth promoting traits

#### PO_4_- solubilizing activity assay.

Pure bacterial colonies were screened for acid production and phosphate solubilizing activity in NBRIP with 0.17M (1% NaCl, 10 g/L) (20 g Glucose, 5.0 g Ca_3_(PO_4_)_2_, 0.1 g (NH_4_)_2_SO_4_, 0.25 g MgSO_4_.7H_2_O, 10 g MgCl_2_.6H_2_O, 0.2 g KCl, 0.1g yeast extract) [69] and for confirmation on Pikovskaya’s agar (per L) (10.0 g Glucose, 5.0 g Ca_3_(PO_4_)_2_, 0.5 g (NH_4_)_2_SO_4_, 0.2 g NaCl, 0.1 g MgSO_4_.7H_2_O, 0.2 g KCl, 0.5 g yeast extract, 0.002 g MnSO_4_.H_2_O, 0.002 g FeSO_4_.7H_2_O), pH 7.0, 15.0 g Agar [70]. A loop of fresh pure colonies of bacteria was spotted on both media. Plates were incubated at 30 ± 2°C for seven days and observed for halo zone formation around bacterial growth. The presence of a halo zone indicated positive phosphate solubilization activity. Sizes of the halo zones were categorized relative to the ones with the largest zones.

#### Zn- solubilizing activity assay.

All halotolerant isolates were inoculated into mineral salts medium (MSM) with 0.17M NaCl as described by Saravanan *et al.* (10.0 g containing dextrose, 1.0 g (NH_4_)_2_SO_4_, 0.2 g KCl, 0.1 g K_2_HPO_4_, 0.2 g MgSO_4_, 1 g ZnO, pH: 7.0; 15.0  g Agar) [71,72], autoclaved at 121°C for 20 min. Plates were incubated at 30 ± 2 °C for seven days and observed for halo zone formation around bacterial growth. The presence of a halo zone indicated positive zinc solubilization activity, categorized as for the phosphate solubilization assay above.

#### IAA production assay.

Indole acetic acid (IAA) production of halotolerant bacteria was determined by inoculating a bacterial colony into LB broth containing 0.17M NaCl with L-tryptophan 0.1% (w/v) and incubated at 30°C at 125 rpm for four days. The broth was then centrifuged at 10,000 rpm for 10 min and 1 mL of supernatant was mixed (1:1 v/v) with Salkowski reagent (98 mL of 35% HClO_4_ and 2 mL of 0.5 M FeCl_3_) and incubated at room temperature [73,74]. IAA was indicated by pink color development and the optical density was recorded at 530 nm after 2 h.

#### Siderophore production assay.

For siderophore production, selected halotolerant strains were examined on the Chrome Azurol’s (CAS) agar medium with 0.17M NaCl [75,76]. The plates were incubated at 30°C for 7 days. Pink color development around the colony was indicative of siderophore production. Relative strength of color was used to categorize the results.

#### Biofilm production assay.

To test whether the halotolerant strains can form biofilms, which may contribute to the plant growth stimulation activity, a 96-well plate crystal violet binding assay was conducted. Biofilm formation was characterized and values measured for each isolate as described previously by optical density (OD) determination at 630 nm [77]. Relative results were categorized as for other assays above.

#### Biolog assay.

We tested halotolerant strains for the BIOLOG assay following instructions outlined in the BIOLOG GEN III MicroPlate^TM^ manual (Biolog Inc., Hayward, CA). Each of the 96 wells contains reagents for a different assay, including redox dyes that are converted to a purple color to indicate positive results. Pure fresh cultures of the selected bacterial isolates were grown on LB agar plates with 0.17M NaCl at 30°C for 48 hours. To ensure proper calibration, we first tested the blank without bacterial inoculation to set the turbidimeter to 100% transmittance. Bacterial colonies were collected using a sterilized wooden stick and mixed with the liquid provided by the BIOLOG GEN III MicroPlate^TM^ kit. The target cell density was adjusted to between 90–98%T. The cell suspension was poured into a multichannel pipette reservoir, and each well in the MicroPlate was filled with 100 µl of the suspension, being careful to not splash from one well into another. The MicroPlate was covered with its lid and incubated at 30°C. Results were recorded as relative intensity of color production in each well after 8 hours and 22 hours of incubation.

#### Minimum inhibitory concentration (MIC) of NaCl for selected isolates.

To evaluate the salt tolerance potential of the halotolerant strains, we determined the minimum inhibitory concentration (MIC) of NaCl for each. Selected strains were inoculated into LB broth and agar plates with 0, 1, 2, 3, or 4 M NaCl. After incubation at 30°C for 48 hours, the NaCl inhibitory concentration was recorded.

### Greenhouse trial of inoculated alfalfa growth promotion under salt stress

All isolates were initially screened on alfalfa plants for the ability to stimulate growth in saline conditions and the ten most promising isolates were selected for further experiments. *Kushneria* strains A3 and B5 were inoculated into alfalfa (Vernal variety, Granite Seeds, Lehi, Utah) to examine growth stimulation in open pots in the presence and absence of salt. Seeds were planted directly into pots containing potting soil (1:1:1 potting soil:clay:sand) and watered with 100 ml of 0.5 × Hoagland’s basic nutrient solution [78] containing 0 for no salt controls or 0.205 M NaCl for salt-treated plants. The initial watering solution contained 1 ml of LB for no salt and no bacteria or 1 ml of liquid LB culture (containing 1 × 10^9^ CFU/ml) for each strain tested. Plants were grown in June 2021 at ambient temperature and light at the Brigham Young University (BYU) Greenhouse Complex with watering every third day with 0.5 × Hoagland’s solution. Plants were harvested after four weeks of growth and analyzed for length of roots, height, and weight.

### Growth chamber trials for alfalfa plant growth stimulation with selected halotolerant bacterial isolates

Alfalfa seeds were surface sterilized with dilute bleach (1% sodium hypochlorite) followed by extensive washing with sterile water and germination in a sterile petri dish. After 24–36 hours the seedlings were transplanted into autoclaved soil (1:1:1 potting soil:clay:sand) in a clear magenta box. One hundred ml of 0.5 × Hoagland’s solution containing 0 for the no salt and no bacteria controls or 0.205M NaCl along with 1 ml of LB broth (for the no bacteria control) or bacterial culture (containing 1 × 10^9^ CFU/ml in LB for each strain) was added to each box. Out of the ten isolates that were initially screened for properties associated with PGP activity, five strains were selected for further trials based on those that had the highest putative plant growth promotion properties and performed the best in the initial growth trials (A3, B5, B2, D8, E4). Five seedlings were transplanted into each of six boxes. A second magenta box was inverted and taped in place to allow air exchange, and the plants were placed in the growth room at 25°C with a 16 hr light/8 hr dark cycle (~200 µmol/m^2^/s). After four weeks of growth, total plant height and weight were measured and analyzed for statistical significance.

### Determination of bacterial survival in alfalfa pots under salt stress conditions

To determine whether the bacterial inoculum could colonize the soil and/or become endophytic in alfalfa roots, soil and root samples were collected when the alfalfa plants were harvested. Soil was diluted in sterile phosphate buffered saline (PBS) and spread on LB agar plates containing 1 M NaCl. Roots were surface sterilized, ground in sterile PBS, and similarly spread on plates. The identification was based on colony characteristics of the bacterial colonies and 16S rRNA gene sequencing as above.

### Genomic integration of a constitutively expressing GFP plasmid into the B5 Strain

The Mobile-CRISPRi system was developed [79] to facilitate the transfer and integration of CRISPR interference (CRISPRi) plasmids into the genome of a wide range of both Gram-positive and Gram-negative bacteria through bacterial conjugation and Tn7 transposition [79,80]. In this system, an integrating plasmid carrying a catalytically inactive Cas9 nuclease (dCas9), single guide RNA (sgRNA) and a helper plasmid are co-transformed into bacteria and integration is selected for using an antibiotic. After integration, the dCas9 can be induced with IPTG to repress expression of the gene targeted by the sgRNA.

We used the Mobile-CRISPRi system to generate a B5 strain that constitutively expresses sfGFP by co-transforming two of the Mobile CRISPRi plasmids pJMP2834 (Addgene Plasmid #160673) and pJMP1039 (Addgene Plasmid #119239). The “test” plasmid pJMP2834 is an integrating plasmid that, in addition to dCas9, constitutively expresses a superfolder green fluorescent protein (sfGFP) and encodes a sgRNA that targets the sfGFP. The helper plasmid pJMP1039 expresses four of the five Tn7 transposition proteins (Tns A, B, C, and D) which insert the integrating plasmid downstream of the highly conserved glmS gene, serving as a chromosomal attachment site known as the attTn7 site [81]. Although bacterial conjugation was possible with the B5 strain, we found that a higher transformation efficiency could be achieved through electroporation of the helper plasmid and the integrating plasmid.

We opted to focus on one strain for this analysis as the A3 and B5 are closely related, and the B5 *Kushneria* strain was selected. B5 cells were made electrocompetent using standard procedures with slight modifications to increase the electroporation efficiency [82]. Briefly, a single colony was picked from a freshly streaked agar plate with *Kushneria* media (LB containing 5% NaCl) and was used to inoculate 5 mL *Kushneria* media which was shaken at 220 rpm at 30°C overnight. 100 ul of the overnight culture was used to inoculate 250 mL Cells were grown to log phase (OD_600_ of 0.4–0.6). The cells were washed 3 times by pelleting the cells at 2000 g for 10 minutes at 4°C and resuspending the cells in 50 mL ice cold 10% glycerol. The cells were then resuspended in 4 mL ice cold 10% glycerol and 50 μL of the electrocompetent cells were mixed with 100 ng of pJMP1039 and 100 ng of pJMP2834 and transferred to a 1 mm electroporation cuvette previously chilled on ice. The cells were then electroporated using a Bio-Rad GenePulser with the following conditions: 1.25 kV, 200 Omega, and 25 μF. Cells were immediately resuspended in 950 μL of room temperature SOC Outgrowth Medium (New England Biolabs) and incubated with shaking at 220 rpm for 2 hours at 30°C before being plated on *Kushneria* media containing 30 mg/mL Kanamycin to select for integration.

This strain was inoculated into alfalfa as above. Upon harvest, root samples were examined using an ECHO Revolve Fluorescence Microscope (Model RVL2-K3) at the BYU Microscopy Center.

### Statistical analyses

All statistical analyses were conducted using R environment 4.1.3 for Windows. The data for shoot length, root length, total plant length, and plant biomass were analyzed through one-way analysis of variance (ANOVA) to identify the significant effects of bacterial treatments on these traits, keeping *P* = 0.05. The R package, Agricolae, was used to carry out Tukey’s Honest Significant Difference (HSD) Post Hoc test to identify which group means differ from each other after finding a significant difference in the overall ANOVA.

## Results

Bacterial isolates were evaluated for the ability to stimulate alfalfa growth in salty conditions. Many were identified as similar or duplicate isolates based on 16S rDNA sequence analysis. Several distinct isolates were analyzed for potential plant growth promotion properties (the source host of each isolate and DNA sequence accession numbers are given in Table 1).

**Table 1:** Source of bacterial strains.

Halophilic strain	Host halophyte	Accession number
A1 *Halomonas* sp.	*A. occidentalis* soil	*NA*
A3 *Kushneria marisflavi*	*A. occidentalis* roots	PP463953
A5 *Bacillus aryabhattai*	*S. utahensis* roots	*NA*
A9 *Halomonas elongate*	*A. occidentalis* roots	MK873884
B1 *Bacillus zanthoxyli*	*S. utahensis* roots	*NA*
B2 *Kushneria* sp.	*S. rubrum* soil	MK873883
B3 *Microbacterium* sp.	*S. rubrum* roots	*NA*
B5 *Kushneria* sp.	*S. utahensis* roots	PP463954
D8 *Bacillus* sp.	*S. utahensis* soil	PP446494
E4 *Halomonas* sp.	*A. occidentalis* soil	PP446495

Accession numbers of 16S rDNA sequences are given for the strains tested for plant growth promotion activity. NA, not available. Host halophytes: *Allenrolfea occidentalis*, *Sarcocornia utahensis* and *Salicornia rubra*.

### PO_4_ and Zn solubilization

**Fig 2:**
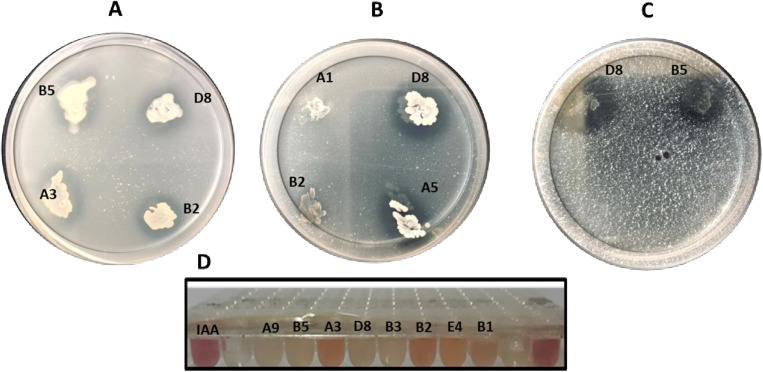
Plant Growth promoting traits of halotolerant bacterial isolates. (A) Pikovskaya’s medium and **(B)** NBRIP medium for phosphate solubilization. (C) mineral salts medium for zinc solubilization. **(D)** IAA production assay in a 96-well plate. Halos around bacteria in A-C indicate positive activity. Results are summarized in Fig 3.

**Fig 3:**
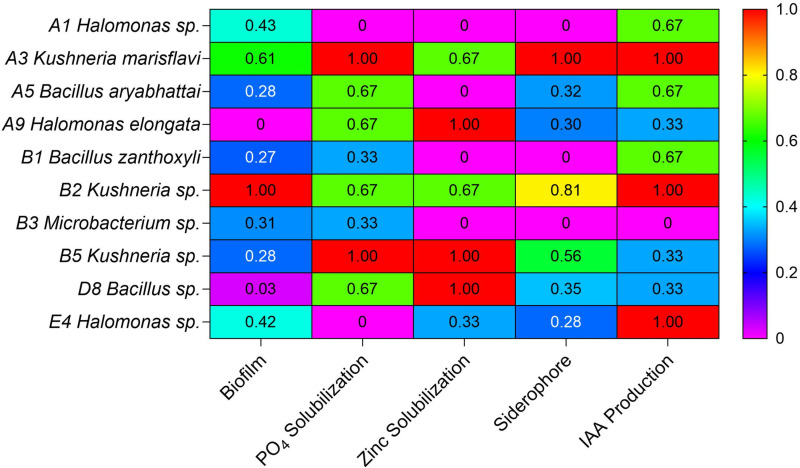
Heat map of putative plant growth promoting bacterial properties. The data was normalized to scale different property values uniformly, ensuring comparability across bacterial strains. Each parameter was transformed to a range between 0 and 1 using min-max normalization, where the minimum observed value was set to 0 and the maximum to 1. The qualitative data indicators (e.g., “-” “+”, “++”, “+++”) were converted into numerical values based on an arbitrary scale before applying min-max normalization, where the minimum observed value was set to 0 and the maximum to 1.

The ten strains were inoculated on NBRIP and Pikovskaya’s agar medium to determine their phosphate solubilizing ability. All strains were grown on both media; however, the image presents a selected subset of bacterial zones (Fig 2). Of these, eight strains showed phosphate solubilizing activity, with A3 and B5 *Kushneria* isolates showing the greatest P solubilization activity among the evaluated strains. Zn solubilization was similarly tested on the appropriate media (see Methods) and the A3, B5 and D8 *Kushneria* strains exhibited the highest activity, with the A3 and B2 strains also showing relatively high activity (Figs 2 and 3).

### IAA production

A3, B2 and E4 strains supported the highest level of IAA production, while A1, A5 and B1 showed relatively high activity, and the other strains showed low or no activity (Figs 2 and 3).

### Siderophore production assay

Siderophores are chelating molecules secreted by microorganisms under low iron stress. They scavenge iron from the environment and form Fe (III)-siderophore complexes, making the otherwise insoluble iron available for bacterial uptake. They play a crucial role in facilitating iron uptake by plants, ultimately promoting their growth and development [83,84]. To test siderophore production activity, strains were spotted on Chrome Azurol’s (CAS) agar medium. Strains A3, B2 and B5 showed the maximum siderophore production, while minimum siderophore production was observed in A5 (*Bacillus* sp.) and A9 (*Halomonas* sp.).

### Biofilm production

The ability to form biofilms has been identified as a potential contributing factor to plant growth promotion, as biofilms can help control nutrient and ion access from the soil to the roots [85]. To test whether the bacterial strains can form biofilms, which may contribute to the plant growth stimulation activity, a plate assay was conducted. The A3 and B2 *Kushneria* isolates showed strong biofilm formation, while other isolates, including the B5 strain, *Bacillus* and *Halomonas* isolates, had lower biofilm activity (Fig 3).

The summary of data shown in Fig 3 shows that the A3, B2 and B5 *Kushneria* strains have high phosphate and zinc solubilization activity, produce IAA (B5 shows lower IAA expression) and have the highest siderophore activity as compared to the *Bacillus* (A5 and B1) and *Halomonas* (A9) isolates. *Kushneria* strains A3 and B2 exhibit the highest biofilm activity.

### Biolog assays

The Biolog plates include different tests in a 96 well plate, allowing screening of strains for the ability to metabolize a variety of sugars and substrates, resistance to antibiotics, and the ability to grow at different salt concentrations or in mildly acidic conditions (pH 6). The results of Biolog analysis of nine strains are shown in S1 Table. The strains exhibit different properties; all can grow in glucose and many of the other sugars. As expected, all nine strains showed growth at 8% NaCl, and most can grow at pH 6 (except the A9 and D8 strains). All except the D8 strain were positive for growth in lithium chloride. Although the A3 and B5 strains are both *Kushneria*, they show differences in utilization of some metabolites (S1 Table). A3 is able to utilize mannose and glucoside, while B5 can utilize neither. Conversely, B5 can metabolize malic acid, citric acid and mucic acid, which A3 cannot. In addition, B5 is resistant to the antibiotics rifamycin and lincomycin, while A3 is not. The data show that these two isolates are distinct from each other. These biochemical properties agree with what is known about other *Kushneria* species [55–59,64].

### Testing of halotolerant strains for NaCl tolerance

**Fig 4:**
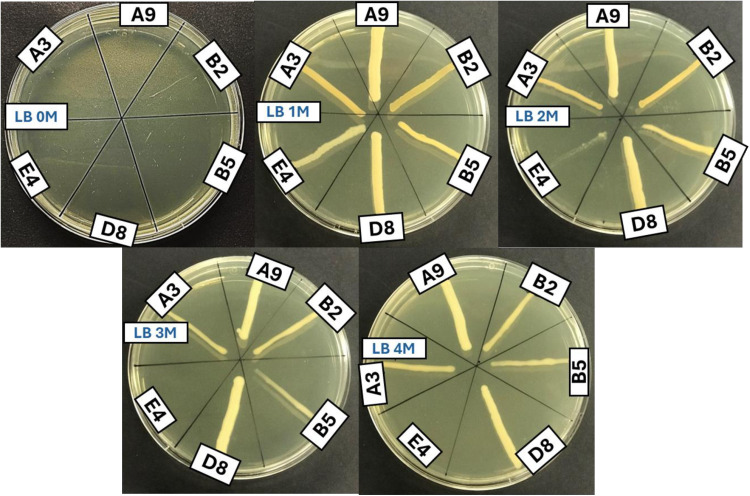
Testing of halotolerant strains on LB agar with 0, 1, 2, 3 & 4 M NaCl. No growth was observed on LB with 0 salt.

As the Biolog plates only provide up to 8% NaCl (1.37 M NaCl) for testing bacteria, selected strains were screened on plates and in liquid media with different NaCl concentrations (0, 1, 2, 3 & 4 M NaCl) to determine their maximum level of salt tolerance. Six strains were found to tolerate 1 M NaCl (A3, A9, B2, B5, D8 & E4). Five of these six strains grew at all concentrations up to 4M NaCl, except for E4 (Fig 4). No growth was observed on plates with no added salt, which indicates that the six strains are halophilic and require salt for growth.

### Greenhouse trials of inoculated alfalfa

**Fig 5:**
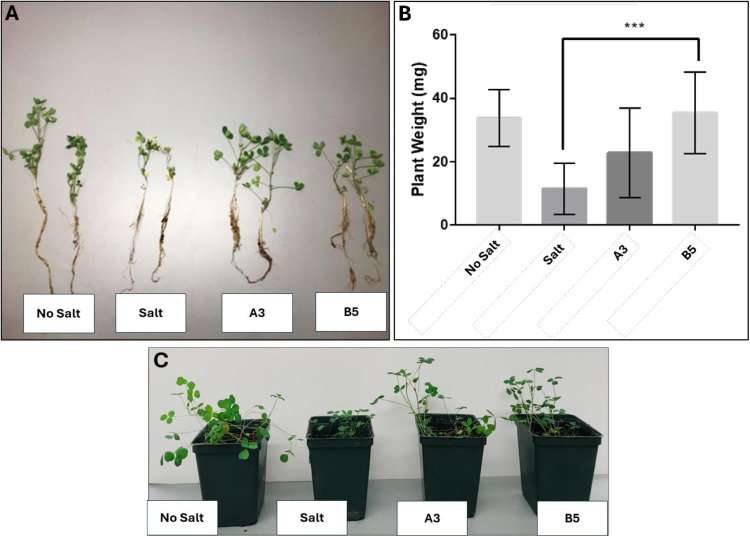
Effect of *Kushneria* strains on alfalfa plant growth in greenhouse open pot trials. (A) photographs of plants after harvesting from each treatment (No salt control without bacteria, Salt control without bacteria, A3 inoculation, B5 inoculation). (B) the total plant fresh weight is shown. Data points indicate means ± SEM of at least 10 biological replicates. The statistically significant difference is marked with asterisks (Tukey’s HSD, P < 0.05). Significance: P < 0.001 ‘***’. (C) Plants in open pots before harvesting.

To examine the growth stimulation potential of the strains under different conditions, alfalfa plants were grown in open pots in the Brigham Young University (BYU) greenhouse with and without inoculation with two *Kushneria* strains (A3 and B5) grown in the presence of 0.205M NaCl. These strains were chosen due to their relatively high, if not highest, levels of properties associated with plant growth promotion activity, including phosphate and zinc solubilization, and biofilm and siderophore production (Fig 3). Clear differences in growth of roots and shoot tissue were observed between the inoculated plants and the control uninoculated plants grown in the presence of salt (Fig 5). The B5 *Kushneria* strain yielded the greatest increase in plant fresh weight. A3, while appearing visually to result in modest enhanced growth, did not show a statistically significant difference (Fig 5B). These two strains were used for more detailed analysis in the growth chamber as individual inoculants and in combination in growth chamber experiments.

### Alfalfa plant growth trial in the growth chamber under salt stress

**Fig 6:**
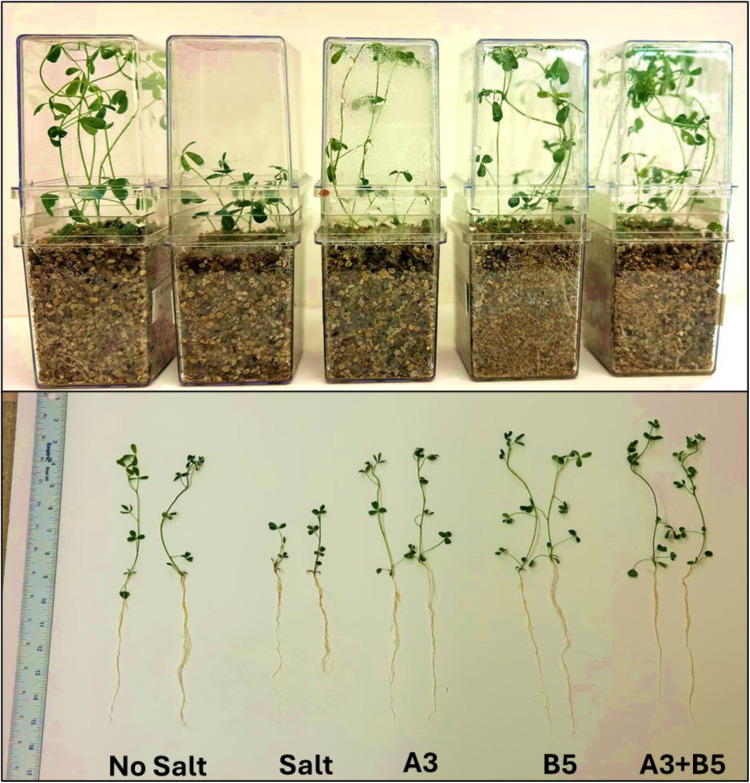
Alfalfa plant growth trial with *Kushneria* strains under salt stress conditions. Harvested alfalfa plants are shown at the bottom. Each pot was inoculated with the indicated bacterial isolate. See Fig 7 for the data analysis.

**Fig 7:**
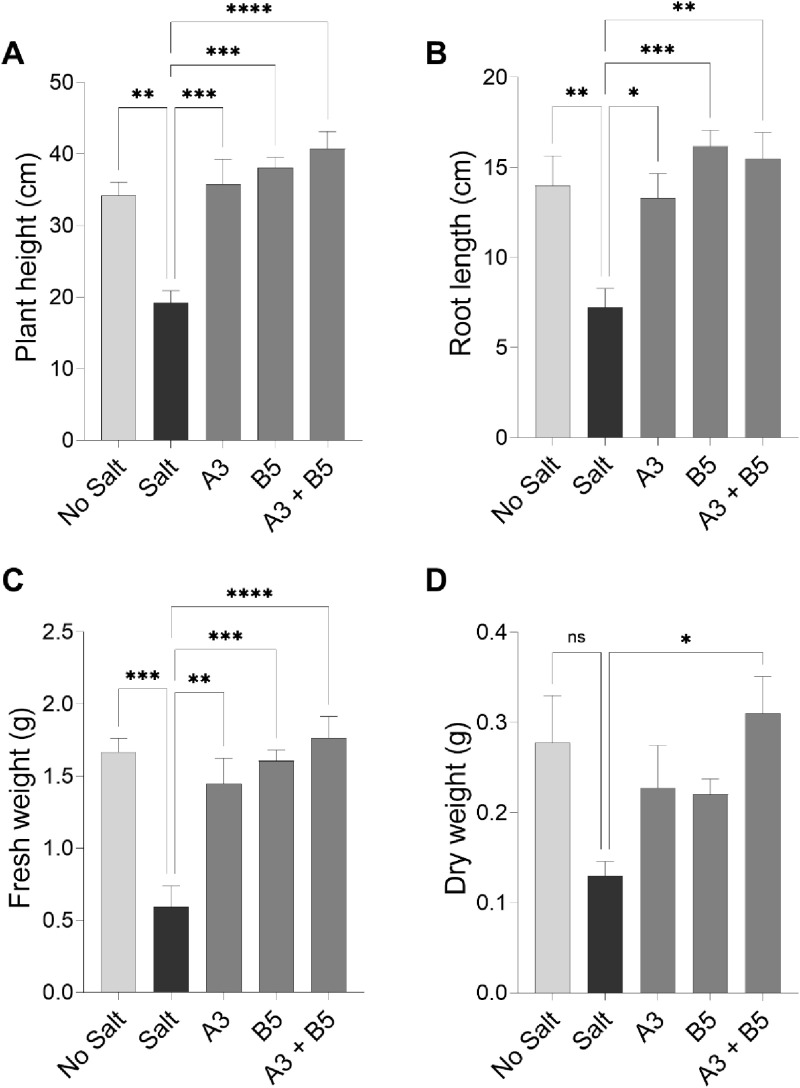
Effect of *Kushneria* strain inoculation on alfalfa in growth chamber trials. (A) plant height, (B) root length, (C) plant fresh weight, and D) plant dry weight. Data points show the means ± SEM of at least 15 biological replicates. Statistically significant differences are marked with asterisks (Tukey’s HSD, *P* < 0.05). Significance codes: 0.001 ‘***’; 0.01 ‘**’; 0.05 ‘*’.

Alfalfa plants were grown with and without inoculation with the *Kushneria* sp. A3, B5 or co-inoculation of A3 and B5 isolates, in the presence and absence of 0.205M (1.2%) NaCl in the watering solution. We selected isolates A3 and B5 for co-inoculation as these two strains produced the best results when tested individually (Figs 6 and 7). Individual isolates A3 and B5 have significant effects on plant height (M = 35.8; 95% CI: -25.74, -7.429 cm *P* = 0.0005 and M = 38.07; 95% CI: -28.01, -9.694 cm *P* = 0.0001), respectively) and root length (M = 13.3; 95% CI: -11.30, -0.8203 cm; *P* = 0.0202 and M = 16.17; 95% CI: -14.16, -3.685 cm; *P* = 0.0009, respectively) in alleviating salt stress. These isolates also increased total plant biomass compared to the plus salt control (Fig 7C, 7D).

We hypothesized that the two strains might contribute to plant growth when inoculated together. We observed that the combination of A3 + B5 resulted in the largest increase in plant height (Fig 7) (M = 40.7; 95% CI: -30.67, -12.35 cm; *P* = 0.0001); it also shows the most reproducible positive results in both plant fresh (M = 1.76; 95% CI: -1.705, -0.6347 g; *P* = 0.0001) and dry weight (M = 0.36; 95% CI: -0.3315, -0.02852 g, *P* = 0.0168), and the B5 strain showed the most promising result for root length (M = 16.17; 95% CI: -14.16 to -3.685 cm, *P* = 0.0009) as compared with others. These findings indicate that the combination of A3 + B5 is the best for promoting alfalfa growth in the presence of 0.205M NaCl as compared to individual isolates.

### Additional trials with other bacterial strains

**Fig 8:**
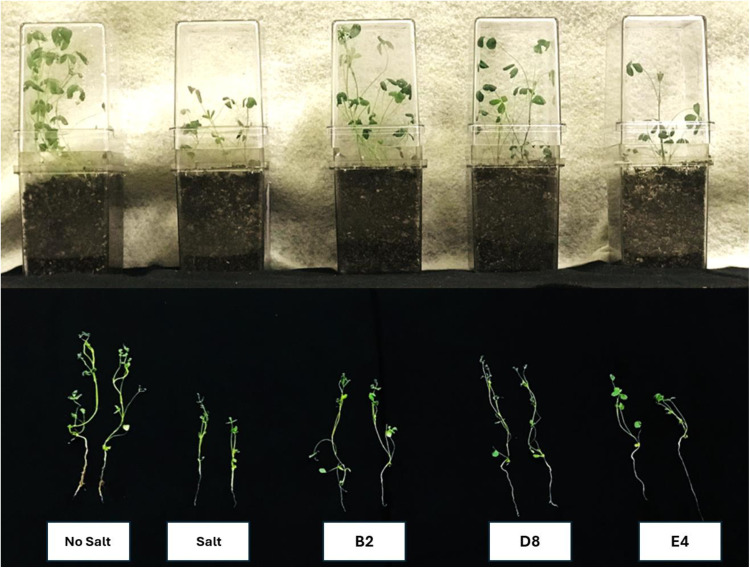
Alfalfa plant growth trial with *Kushneria* strains under salt stress conditions (top panel). Harvested alfalfa plants (bottom panel). All plants were grown in closed pots with a single watering upon planting and inoculated with the indicated bacterial isolate except for the controls (uninoculated No salt & salt). See Fig 9 for analysis of plant length and weight measurements.

**Fig 9:**
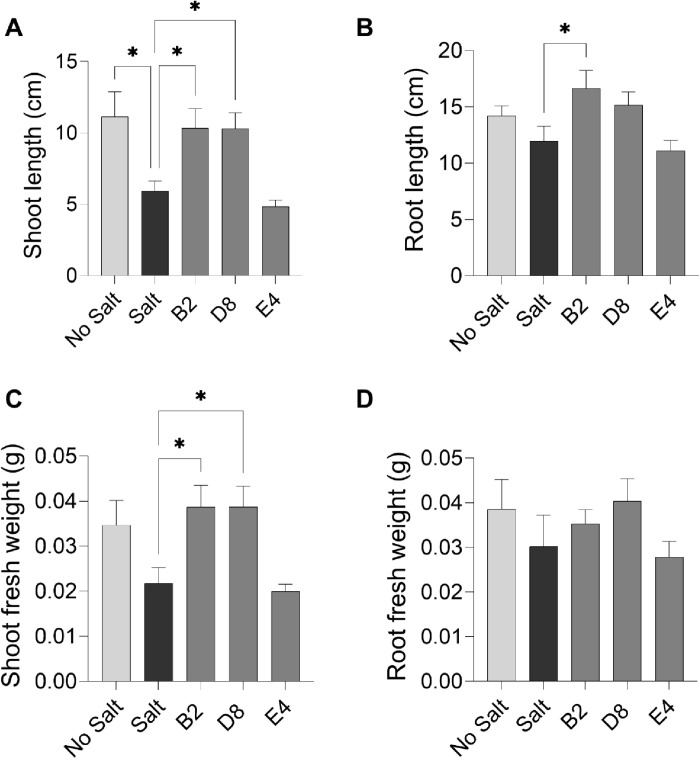
Effect of *Kushneria* strain on alfalfa plants. (A) shoot length, (B) root length, (C) shoot fresh weight, and (D) root fresh weight. Data points show the means ± SEM of at least 13 biological replicates except for E4 which has 10 replicates and the unstressed control which has 4 replicates. Statistically significant differences are marked with asterisks (Tukey’s HSD, P < 0.05). Significance codes: 0.001 ‘***’; 0.01 ‘**’; 0.05 ‘*’.

Three other strains were tested for plant growth promotion activity. Alfalfa plants were grown with and without inoculation with the *Kushneria* strain B2, *Bacillus* strain D8, and *Halomonas* strain E4 in the presence and absence of 0.205M NaCl in the watering solution. Results are shown in Figs 8 and 9 (harvested plants and data analysis of shoot length, root length, shoot and root fresh weight). While the B2 and D8 strains showed some promise for plant growth stimulation, similar to A3 and B5 (Figs 8 and 9), other strains appeared to be inhibitory or had little effect on plant growth (not shown). Strains B2 and D8 stimulated growth (shoot length) in the presence of 0.205M salt (M = 10.33 (95% CI: -8.110 to -0.6976 cm, *P* = 0.0135), and M = 10.33 (95% CI: -8.091, -0.6784 cm; *P* = 0.0140), respectively. However, *Halomonas* strain E4 inhibited plant growth when compared to other isolates (M = 4.82; 95% CI: -2.876, 5.073 cm; *P* > 0.05). The *Kushneria* B2 strain showed the highest growth stimulation for root length compared to other isolates (M = 16.63; 95% CI: -9.171, -0.2141 cm, *P* = 0.03), and D8 and E4 did not show any significant effect on root length (M = 15.15; 95% CI: -7.690, 1.267 cm; *P* = 0.2897 and M = 11.10; 95% CI: -3.960, 5.645 cm; *P* = 0.999, respectively). The B2 and D8 strains supported the greatest promotion of shoot fresh weight in this experiment. These strains also show reproducible but statistically not significant increases in root fresh weight (*P* = 0.4570 and *P* = 0.9999, respectively (Fig 9). Strain E4 appeared to have an inhibitory effect or no effect on plant shoot (M = 0.01; 95% CI: -0.01360 to 0.01718 g, *P* > 0.05) and root fresh weight (M = 0.02; 95% CI: -0.01855, 0.02334 g; *P* > 0.05).

### Confirmation of re-isolated colonies from alfalfa plants

**Fig 10:**
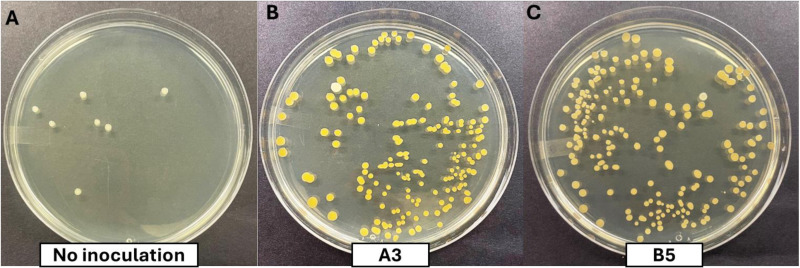
Survival determination of inoculated bacteria recovered from alfalfa grown in pots. Bacteria from crushed root tissue after harvest were grown on LB medium containing 1M NaCl with 10^-6^ dilution after harvesting. (A) No inoculation (B) A3 inoculation (C) B5 inoculation.

Since the A3 and B5 strains showed the greatest stimulatory effect, root tissue and soil from around the roots of inoculated plants were harvested, ground in buffer, and plated to recover bacteria. Uninoculated soil and roots were used as controls. Colonies recovered included the same bacteria used to inoculate the plants. However, contaminating bacteria were also obtained, including mostly *Bacillus* species. Even though autoclaved soil was used in the growth chamber trials, it was difficult to sterilize the soil completely, which is potentially the cause of the observed contamination (Fig 10). The *Kushneria* strains have a distinctive orange pigment that aids in identification, which was confirmed by partial 16S rRNA gene sequencing.

### Visualization of B5 expressing GFP in inoculated plants

**Fig 11:**
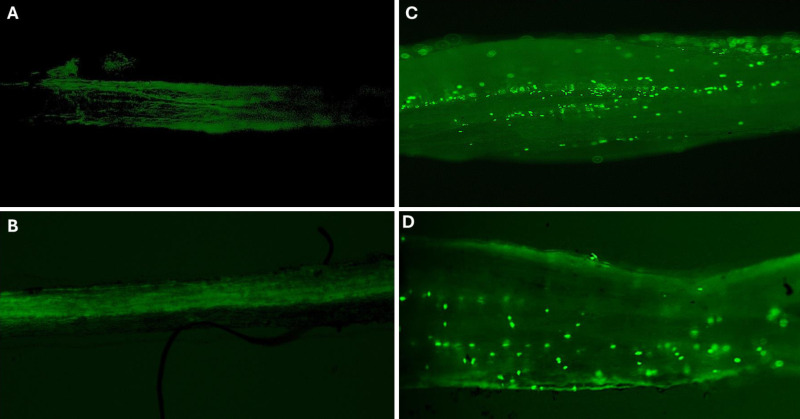
Visualization of B5 strain expressing GFP in alfalfa root tissue. (A & B) No inoculation, (C & D) B5 + GFP inoculation. Root sample from alfalfa inoculated with B5 strain, constitutively expressing the GFP gene as compared to control, visualized using the Echo Revolve microscope. Fluorescent GFP signal highlights bacterial colonization within the root tissue.

Inoculation with the B5 *Kushneria* strain carrying a constitutively expressing GFP gene allowed visualization of bacterial colonization within the root tissue of alfalfa plants (Fig 11). The root samples were examined using an ECHO Revolve Fluorescence Microscope (Model RVL2-K3), which provided high-resolution images of GFP-expressing bacteria in the plant root tissue. Fig 11 compares root tissues inoculated with the B5 strain and control root tissues that were not inoculated. In the GFP-inoculated samples, the B5 strain is visible as bright green fluorescent punctae (Fig 11C, 11D), indicating successful colonization and persistence of the bacteria within the plant roots. Only background fluorescence was observed in the control non-inoculated root samples (Fig 11A, 11B).

## Discussion

The challenge of producing adequate food for the growing world population is complicated by many biotic and abiotic factors. One serious problem is soil salinity, which affects many current agricultural areas and is increasing due to climate change and poor irrigation practices. The accumulation of surplus sodium and chloride ions, which are toxic in excess, impedes essential metabolic pathways and compromises the plant’s homeostatic equilibrium. In work carried out by others, as detailed in the introduction, some rhizobacteria have been found that can promote plant growth in the presence of salt. We initiated this research to extend our earlier work [68] and identify salt-tolerant bacterial strains with the potential for inoculation of salt-sensitive crops to increase yield in salt-affected soils.

In this current study we identified two strains of *Kushneria* that each stimulate alfalfa growth in salty soil conditions under controlled conditions in pots. When inoculated together in young alfalfa seedlings, the A3 and B5 strains showed an enhanced effect on plant growth. The analysis of biochemical activities of these strains and eight others, including additional *Kushneria* isolates and *Bacillus* and *Halomonas* strains, led to the identification of potentially important traits of the strains that most effectively stimulate alfalfa growth in the presence of 0.205 M (1.2% NaCl) in the watering solution. In particular, the A3 and B5 strains showed relatively high activity for phosphate and zinc solubilization and siderophore production. Both produce IAA, with A3 showing higher activity than B5. Both exhibited biofilm formation, while A3 again was higher. The B2 *Kushneria* strain was also high for these activities and appears to be related to the A3 strain based on rDNA sequence analysis and some properties (Fig 3), while there are some differences in utilization of some carbohydrate sources (S1 Table). The A3 strain was able to grow with mannose and glucoside, while B5 could not. The B5 strain grew with malic acid, mucic acid, and citric acid, while A3 could not. In addition, there were differences in susceptibility to some antibiotics. Collectively, the differences in properties indicate that the A3 and B5 isolates are distinct strains of *Kushneria*.

When alfalfa plants were exposed to NaCl stress without added bacteria, a notable reduction in their growth was observed (Figs 5–8, salt-stressed plants), with more pronounced effects on roots. Uninoculated plants grown in the presence of salt were smaller with less stem and root branching, and fewer leaves per plant compared to control plants grown in the absence of salt or the plants inoculated with A3, B5 or both (Figs 5, 6, and 8). The higher inhibition by NaCl stress on roots is plausible due to their direct contact with a higher salt concentration in the soil [86]. In some cases it has been suggested that water absorption may lead to the enhanced root growth [87].

There is a growing number of publications on plant growth promotion by salt-tolerant (halotolerant) rhizobacteria associated with halophyte species in saline soils. Some of these bacteria are closely associated with the roots, while others are established within plant tissues (endophytes). Mechanisms by which these bacteria enhance plant growth have been proposed to include enhanced nutrient acquisition and changes in bacterial and host plant gene expression [49,88,89]. *Burkholderia phytofirmans* is an endophyte that alters the expression of plant transcription factors known to regulate the expression of plant stress genes [90]. Some bacterial endophytes (S*phingomonas, Pantoea, Bacillus* and *Enterobacter)* have been reported to enhance the salt tolerance of hybrid elephant grass [91].

The genus *Kushneria* has been characterized as a plant growth-promoting rhizobacteria (PGPR) known to produce Indole-3-acetic acid (IAA), biofilm, and siderophores, as well as solubilize zinc and phosphate [92]. In our work, we observed a differential growth response to NaCl stress when plants were inoculated with different bacterial strains. Four *Kushneria* isolates (A3, B5, B2 and D8) exhibited the most significant effects. The varying performance of these strains may be linked to the differential production of IAA, with the A3 *Kushneria* strain exhibiting the highest IAA levels, more than B5 or other isolates. A3 was highest in three of the tested properties, and relatively high for the other two properties (Fig 3). B5 also showed relatively high activity for all five properties (Fig 3). Further investigation is needed to elucidate the correlation between plant growth promotion and IAA, especially considering that certain strains in our study, such as E4, produced IAA but did not support growth stimulation in plant trials. As the main auxin in plants, IAA plays a crucial role in modulating plant growth by influencing cell division, stimulating stem elongation, and enhancing root branching through increased cell wall synthesis. The observed variations in plant growth responses to IAA-producing strains might also be influenced by factors such as the concentration of IAA produced, the timing of its release, and the specific mechanisms through which these strains interact with plant tissues. IAA-producing bacterial strains *P. aureantiaca* and *P. extremorientalis* were found to alleviate the reductive effect of salt stress on percentage of germination [93]. Another study showed that *Kushneria marisflavi*, in combination with *Pseudomonas stutzeria*, reduced salinity stress-induced damage in barley, sunflower and lettuce [43].

Both the A3 and B5 *Kushneria* strains, along with the D8 strain, had relatively high phosphate and zinc solubilization activity compared to other isolates. This bacterial process releases organic acids into the soil, important for phosphorus cycling, leading to the solubilization of phosphate complexes [94] to ortho-phosphate, a form readily available for plant uptake and utilization [95]. Other studies have shown that some bacteria exhibit enhanced solubilization of inorganic phosphate in the presence of NaCl [96,97]. However, it is also reported that there is a decrease in phosphate solubilization activity in certain stress-tolerant phosphate-solubilizing strains at salt concentrations exceeding 0.4 M [98]. In our study, all tested strains demonstrated a high phosphorus-solubilizing ability even in the presence of 1 M NaCl, except for A1 and E4. Halophile and halotolerant microorganisms with phosphate-solubilizing capabilities have exhibited the potential to enhance crop yield under salt stress [99].

Zinc, essential for plant development, was investigated for its solubilization by the selected bacterial strains. All tested strains exhibited zinc solubilization activity associated with plant growth promotion except A1, A5, B1 and B3. This observation is consistent with findings from previous studies that demonstrated that microbes can influence the uptake of toxic ions and nutrients by modulating host physiology or directly reducing the accumulation of Na^+^ and Cl^−^, while concurrently increasing the uptake and translocation of other cations such as zinc [100]. However, the exact mechanisms underlying these processes remain unknown [101,102]. In our study, strains A3, B5, and D8 demonstrated significant zinc solubilization activity. Earlier research has shown that applying PGPR can enhance zinc translocation to rice and wheat [103,104].

**Fig 12:**
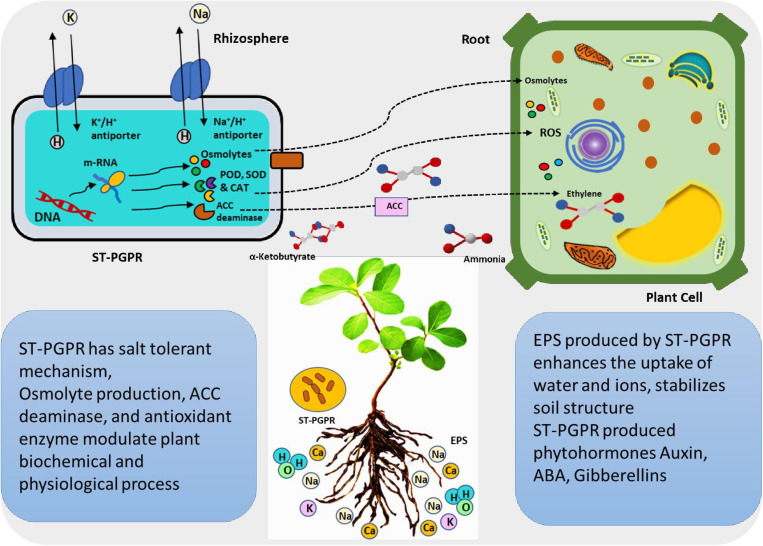
Potential mechanisms for salt-tolerant plant growth promoting rhizobacterial mitigation of salt stress in plants. Concept from [26].

Of the strains tested, A3 and B2 showed the highest biofilm formation, while the B5 strain was considerably lower (Fig 3). Biofilm-producing bacteria have been shown to enhance soil structure, increase soil porosity and facilitate water and nutrient movement from soil to plant [92]. The biofilm structures, characterized by extracellular polymeric substances (EPS), adhere to soil particles, serving as an effective matrix for soil moisture retention. Biofilms not only protect roots from desiccation but also mitigate the impact of salt stress by sequestering toxic Na^+^ and Cl^−^ ions [105,106]. However, from our observations it appears that not all bacteria exhibiting high biofilm production necessarily promote plant growth under salt stress. A summary of potential bacterial growth promotion properties and their effects on plant growth is shown in Fig 12.

Salinity affects various growth parameters of *Lactuca sativa*, including the number of leaves and plant fresh weight [107]. In our study we observed a salinity effect on alfalfa growth parameters, as plant height and mass were compromised without inoculation of bacteria compared to plants with bacterial inoculation under salt stress. Different species of plants may exhibit considerable differences in salinity tolerance in response to inoculation with halotolerant bacteria. The properties of each bacterial strain may have different effects on various plant hosts. The intricate interplay between inoculation, salinity levels, and plant species-specific responses underscores the complexity of plant-microbe interactions in the context of salt stress.

One notable observation during this study was that, in the context of salt stress, the inoculation of plants with both A3 and B5 strains resulted in a notable additive increase in growth parameters, including shoot length, root length, and fresh weight, as shown in Figs 5 and 7. Conversely, the E4 strain significantly reduced these growth parameters, indicating that not all strains have ST-PGP activity. This halotolerant strain appears unable to ameliorate the stress effectively.

While this study focused on the direct effects of salt-tolerant bacterial inoculation on alfalfa growth under salt stress, this was done in controlled laboratory and greenhouse experiments, and plants were not grown to maturity or to the stage where they produce seeds. As such, we did not investigate the broader impacts on the microbiome structure and function associated with plants in a field setting. Future research should explore challenges to the use of halotolerant bacteria strains including how they interact with the native microbial community, potential shifts in community composition, functional dynamics, and their combined contributions to plant health and stress resilience [108]. Understanding these factors could provide valuable insights into optimizing microbial inoculants for sustainable agriculture.

This work provides promising support for using halotolerant bacterial strains, initially isolated from halophytes, as inoculants of crop plants to stimulate growth in salty soil conditions. The A3 and B5 *Kushneria* strains have shown encouraging results with alfalfa, and we have initiated testing of these strains with other plant species including rice, corn, and wheat grass. Further studies are needed to identify the optimal bacterial isolates and combinations for each crop plant. One of the next critical steps will be to conduct field experiments to determine the effectiveness of inoculation with these *Kushneria* strains in competition with native bacteria and examine the persistence of these introduced bacteria in the soil.

## Conclusions

Several halotolerant strains of bacteria isolated from halophytes were found to have varying levels of potential plant growth promotion properties, including phosphate and zinc solubilization, as well as IAA, siderophore and biofilm production. The A3 and B5 *Kushneria* strains exhibited promising results for the stimulation of growth of alfalfa in the presence of salt concentrations that mimic salinity levels often found in affected soils. We have observed the most consistent results in greenhouse and growth chamber trials with *Kushneria* strains A3 and B5. The combination of A3 and B5 showed an increase in growth stimulation than either strain alone. This study was conducted without inoculation of alfalfa with its nitrogen-fixing symbiont *Sinorhizobium meliloti* to determine the effect of the inoculated ST-PGP strains alone in the presence of salinity. The next step to introduce *S. meliloti* to measure the effects of combinations of bacteria, including *S. meliloti*, on alfalfa growth under salty conditions is currently in progress. In addition, further research to examine the potential of these strains under field conditions and with different crop species is warranted. The results presented here provide a strong foundation for the use of halotolerant *Kushneria* for the development of inoculants, which will provide farmers with an additional approach to deal with decreased crop yields caused by soil salinity.

## Supporting information

S1 Table Biolog test for selected salt-tolerant and halophilic bacterial isolates. (DOCX)

S2 Raw Data Raw data file for bar graph figures. (XLSX)
