# Supplementary material for: Characterization of halotolerant Kushneria isolates that stimulate growth of alfalfa in saline conditions
Source: PLoS One. 2025 May 7;20(5):e0322979. doi: 10.1371/journal.pone.0322979 (PMC12057942; doi:10.1371/journal.pone.0322979)
Supplement: S1 Table — (DOCX) [file pone.0322979.s001.docx]

Table 2: Biolog test for selected salt-tolerant and halophilic bacterial isolates

| **BIOLOG Test** | **A1** | **A3** | **A5** | **A9** | **B1** | **B2** | **B5** | **D8** | **E4** |
| --- | --- | --- | --- | --- | --- | --- | --- | --- | --- |
| Dextrin | - | - | - | ++ | + | ++ | - | ++ | ++ |
| D-Maltose | ++ | - | - | ++ | + | ++ | - | ++ | ++ |
| D-Glucose | ++ | ++ | ++ | ++ | + | ++ | ++ | ++ | ++ |
| D-Mannose | ++ | ++ | - | ++ | + | ++ | - | + | ++ |
| D-Fructose | ++ | ++ | - | ++ | + | ++ | ++ | + | ++ |
| D-Galactose | ++ | ++ | - | ++ | + | ++ | ++ | + | ++ |
| D-Mannitol | ++ | - | ++ | ++ | + | + | - | ++ | ++ |
| D-Sorbitol | ++ | - | ++ | ++ | - | + | - | ++ | + |
| D-Trehalose | ++ | ++ | - | ++ | + | ++ | ++ | - | + |
| D-Cellobiose | ++ | - | - | ++ | - | ++ | - | + | + |
| D-Lactose | ++ | - | - | ++ | - | ++ | - | + | ++ |
| D-Melibiose | ++ | - | - | ++ | ++ | ++ | - | + | ++ |
| D-Glucoside | ++ | ++ | - | ++ | + | ++ | - | - | + |
| D-Turanose | ++ | - | - | ++ | - | ++ | - | ++ | - |
| D-Saccharic acid | ++ | + | - | ++ | - | + | ++ | ++ | ++ |
| D-Gluconic acid | ++ | ++ | + | ++ | + | ++ | ++ | + | + |
| D-Galacturonic acid | ++ | + | - | ++ | ++ | ++ | ++ | ++ | ++ |
| D-Malic acid | ++ | - | ++ | ++ | ++ | ++ | ++ | + | + |
| Mucic acid | ++ | - | - | ++ | ++ | + | ++ | ++ | - |
| Lactic acid | ++ | + | - | ++ | ++ | ++ | + | + | ++ |
| Citric acid | + | - | - | ++ | - | ++ | ++ | ++ | + |
| Acetic acid | ++ | - | - | ++ | + | ++ | + | ++ | ++ |
| Formic acid | - | ++ | - | ++ | - | ++ | ++ | ++ | ++ |
| Propionic acid | + | - | - | ++ | - | - | - | ++ | ++ |
| Sodium butyrate | - | - | - | ++ | ++ | - | - | - | + |
| Bromo-Succinic acid | ++ | + | - | + | - | + | ++ | ++ | ++ |
| Lithium chloride | ++ | ++ | ++ | + | ++ | + | ++ | - | ++ |
| Amino-Butyric acid | ++ | ++ | - | ++ | - | + | + | ++ | ++ |
| Vancomycin | ++ | + | - | - | ++ | - | ++ | - | - |
| Glucuronamide | ++ | + | - | - | ++ | + | + | ++ | + |
| L-Galactonic acid lactone | ++ | + | - | + | ++ | ++ | ++ | ++ | - |
| L-Arginine | ++ | - | + | ++ | - | ++ | - | + | + |
| L-Aspartic acid | ++ | - | - | ++ | - | ++ | - | ++ | ++ |
| L-Glutamic acid | ++ | ++ | - | ++ | - | + | ++ | ++ | + |
| L-Histidine | ++ | - | - | ++ | - | + | - | + | - |
| L-Serine | ++ | + | + | - | - | - | ++ | ++ | + |
| L-Malic acid | ++ | - | - | ++ | + | ++ | ++ | ++ | + |
| Lincomycin | - | - | ++ | - | + | - | ++ | - | + |
| Rifamycin SV | ++ | - | ++ | - | ++ | + | ++ | - | + |
| Methyl pyruvate | ++ | ++ | - | + | - | ++ | ++ | + | + |
| 1% Sodium Lactate | ++ | ++ | ++ | ++ | ++ | ++ | ++ | - | + |
| Gentiobiose | ++ | + | - | + | + | ++ | - | ++ | + |
| Neuraminic acid | ++ | - | - | ++ | - | ++ | - | ++ | - |
| Sucrose | ++ | + | - | ++ | - | ++ | ++ | ++ | ++ |
| Tetrazolium Violet | + | - | - | - | ++ | - | + | - | + |
| Stachyose | + | - | - | + | - | ++ | - | ++ | + |
| pH 6 | ++ | ++ | ++ | - | ++ | ++ | ++ | - | ++ |
| 1% NaCl | ++ | ++ | ++ | ++ | ++ | ++ | ++ | ++ | ++ |
| 4% NaCl | ++ | ++ | ++ | ++ | ++ | ++ | ++ | ++ | ++ |
| 8% NaCl | ++ | ++ | ++ | ++ | ++ | ++ | ++ | ++ | ++ |

See Table 1 for identification of strains. Symbols: ++, strong color and positive reaction;

+, slight reaction with light color; -, no reaction or color
